# Does Intraoperative Blood Loss Affect the Short-Term Outcomes and Prognosis of Gastric Cancer Patients After Gastrectomy? A Meta-Analysis

**DOI:** 10.3389/fsurg.2022.924444

**Published:** 2022-06-14

**Authors:** Ze-Lin Wen, Da-Chun Xiao, Xiong Zhou

**Affiliations:** Department of Gastrointestinal Surgery, Chongqing Medical University, Yongchuan Hospital, Chongqing, China

**Keywords:** intraoperative blood loss, gastric cancer, surgery, prognosis, outcomes

## Abstract

**Purpose:**

The purpose of the current meta-analysis was to analyze whether intraoperative blood loss (IBL) influenced the complications and prognosis of gastric cancer patients after gastrectomy.

**Methods:**

We systematically searched the PubMed, Embase and Cochrane library databases on November 29, 2021. The Newcastle-Ottawa scale was used to evaluate the quality of included studies. This meta-analysis uses RevMan 5.3 for data analysis.

**Results:**

A total of nine retrospective studies were included in this meta-analysis, involving 4653 patients. In terms of short-term outcomes, the Larger IBL group has a higher complication rate (OR = 1.94, 95% CI, 1.44 to 2.61, *P* < 0.0001) and a longer operation time (OR = 77.60, 95% CI, 41.95 to 113.25, *P* < 0.0001) compared with the smaller IBL group, but the Larger IBL group had higher total retrieved lymph nodes (OR = 3.68, 95% CI, 1.13 to 6.24, *P* = 0.005). After pooling up all the HRs, the Larger IBL group has worse overall survival (OS) (HR = 1.80, 95% CI, 1.27 to 2.56, *P* = 0.001) and disease-free survival (DFS) (HR = 1.48, 95% CI, 1.28 to 1.72, *P* < 0.00001).

**Conclusion:**

Larger IBL increased operation time and postoperative complications, and decreased OS and DFS of gastric cancer patients. Therefore, surgeons should be cautious about IBL during operation.

## Introduction

Gastric cancer (GC) is currently the fourth largest malignant tumor worldwide and the second largest cause of cancer-related deaths in the world (1, 2). There are various treatments for GC, among which radical gastrectomy is the essential method (3–7). The surgical methods include total gastrectomy and partial gastrectomy, accompanied by lymph node dissection (8).

The influence of intraoperative blood loss (IBL) on the short-term outcomes and long-term prognosis after surgery is concern for surgeons (9). Studies reported that the amount of IBL was associated with colorectal cancer and pancreatic cancer after surgery (9–11).

There has been controversy about the influence of IBL on the short-term outcomes and long-term prognosis of GC patients after gastrectomy (12–19). Some studies reported that IBL did not affect the prognosis (12), while some studies reported that larger IBL had an adverse effect on the outcomes of GC patients (13–19). Therefore, the purpose of this meta-analysis was to explore whether IBL influenced the complications and prognosis of GC patients after gastrectomy.

## Methods

This meta-analysis was conducted on the basis of the preferred reporting items for systematic reviews and meta-analyses (PRISMA) guidelines (20).

### Search Strategy

Two researchers independently searched the English literature in PubMed, Embase and Cochrane Library databases, and the search date was November 29, 2021. The search items were as follows: (“blood loss” OR “intraoperative blood loss”) AND (“gastric cancer” OR “gastric carcinoma” OR “gastric neoplasms” OR “stomach cancer” OR “stomach carcinoma” OR “stomach neoplasms”). In addition, we also searched the references of all included articles to avoid omissions.

### Inclusion and Exclusion Criteria

The inclusion criteria were as follows: 1. Original study; 2. The patients were pathologically diagnosed with GC and underwent surgical treatment; 3. There was a comparison between the smaller intraoperative blood loss (SIBL) group and the larger intraoperative blood loss (LIBL) group in the study; 4. The results of the study included at least short-term outcomes or long-term prognosis. The exclusion criteria included: 1. Non-original studies such as reviews, meeting, comments, case report, etc.; 2. Short-term outcomes and long-term prognostic were insufficient.

### Data Extraction

The data of the included studies were extracted as follows: 1. Study information included first author, country, publication year, study design, IBL definition, sample size; 2. Baseline information included sex, age, BMI, adjuvant chemotherapy, tumor size, tumor staging and tumor differentiation; 3. Surgical information included operation time, the scope of resection and the number of lymph node dissection; 4. Prognosis included long-term prognosis such as overall survival (OS) and disease-free survival (DFS). The data were independently extracted and cross-checked by two reviewers, and if there was a disagreement, the group discussion resolved it.

### Quality Assessment

Two reviewers used the Newcastle-Ottawa Scale (NOS) to assess the quality of the included studies independently (21). The NOS evaluates the quality of studies based on three categories (selection, comparability, and results). The scores were 0–9, of which 9 were high-quality studies, 7–8 were medium-quality studies, and other scores were low-quality studies. If there were differences in the evaluation process, the group discussion resolved it.

### Statistical Analysis

Categorical variables were analyzed with 95% confidence interval (CI) odds ratio (OR); continuous variables were analyzed with 95% CI mean difference (MD); and survival variables were analyzed with 95% CI hazard ratio (HR) for analysis. Among them, HR was extracted from multivariate analysis and/or univariate analysis or estimated from the Kaplan-Meier survival curve (22, 23). The heterogeneity of each study was evaluated by I^2^ and Chi-square test: I2 > 50% indicated high heterogeneity, using random effects model, *P* < 0.1 was considered statistically significant; I^2^ ≤ 50% was using fixed effects model, *P* < 0.05 was considered statistically significant (24, 25). This meta-analysis used RevMan 5.3 (Cochrane Collaboration, London, United Kingdom) for data analysis.

## Results

### Study Selection

**Figure 1:**
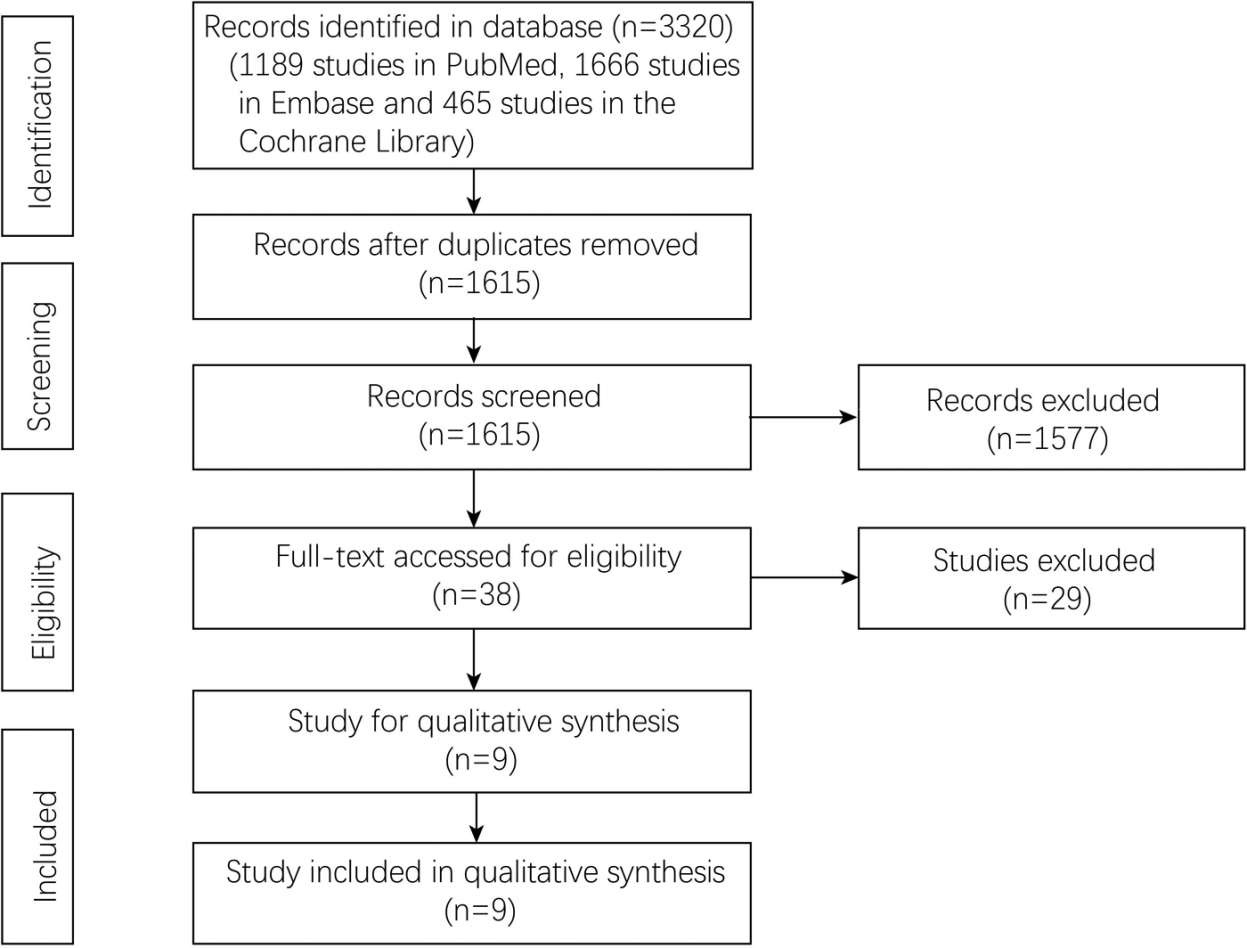
Flowchart of study selection.

A total of 3320 studies were included through the initial literature search. After deleting 1705 duplicate studies, 1615 studies remained. And 1577 studies were excluded through the screening of titles and abstracts. After evaluating the full texts of the remaining 38 studies, according to the inclusion and exclusion criteria, nine studies (12–19, 26) published from 2000 to 2021 were finally included. The screening process was shown in Figure 1.

### Study Characteristics

This meta-analysis included nine relevant studies with a total of 4653 patients including 1392 patients in the LIBL group and 3261 patients in the SIBL group. Nine studies were retrospective studies, of which seven were from Japan and the remaining two were from China and American. The publishing year was from 2000 to 2021 and the study date was from 1979 to 2016. The cut-off value was according to the transfusion, 330, 400, 500 and 990 mL. The specific information of the included studies and the NOS score were summarized in Table 1.

**Table 1:** Characteristics of the studies included in the meta-analysis.

Author	Year published	Country	Study design	Study date	Sample size			Definition of larger IBL and smaller IBL		NOS
					Larger IBL group	Smaller IBL group	Total	Larger IBL group	Smaller IBL group	
Hayashi M (13)	2021	Japan	Retrospective	2008–2015	26	89	115	IBL > 990	IBL ≤ 990	7
Tamagawa H (14)	2020	Japan	Retrospective	1995–2016	42	80	122	IBL ≥ 400	IBL < 400	6
Zhao B (12)	2019	China	Retrospective	1999–2011	577	1092	1669	IBL ≥ 400	IBL < 400	8
Ito Y (15)	2019	Japan	Retrospective	2010–2014	201	305	506	IBL > 330	IBL ≤ 330	8
Mizuno A (16)	2016	Japan	Retrospective	1999–2015	71	132	203	IBL ≥ 400	IBL < 400	7
Kanda M (26)	2016	Japan	Retrospective	1999–2014	57	193	250	With transfusion	Without transfusion	7
Squires MH 3rd (17)	2015	American	Retrospective	2000–2012	168	597	765	Transfused	Non-transfusion	8
Ojima T (18)	2009	Japan	Retrospective	1991–2002	154	702	856	Transfused	Non-transfusion	8
Dhar DK (19)	2000	Japan	Retrospective	1979–1989	96	71	167	IBL > 500	IBL ≤ 500	6

*Abbreviations: IBL, intraoperative blood loss, mL; NOS, Newcastle-Ottawa Scale.*

### Baseline Information

The baseline information included sex, age, body mass index (BMI), adjuvant chemotherapy, tumor size, tumor T stage, tumor N stage and tumor differentiation. Through analysis, we found that the LIBL group had more males (OR = 2.30, 95% CI, 1.17 to 4.53, *P* = 0.02), lower BMI (OR = 0.77, 95% CI, 0.31 to 1.22, *P* = 0.0009), more tumors located in the upper stomach (OR = 2.13, 95% CI, 1.65 to 2.76, *P* < 0.00001) and larger tumors (Tumor size ≤5 cm: OR = 0.66, 95% CI, 0.55 to 0.78, *P* < 0.00001; Tumor size >5 cm: OR = 1.52, 95% CI, 1.28 to 1.81, *P* < 0.00001), more T3-4 (OR = 1.58, 95% CI, 1.30 to 1.91, *P* < 0.00001) and more N1-3 (OR = 1.43, 95% CI, 1.16 to 1.75, *P* = 0.007). In addition, there was no significant difference between the two groups in terms of age (OR = 0.22, 95% CI, −1.59 to 2.04, *P* = 0.81), adjuvant chemotherapy (OR = 1.35, 95% CI, 0.59 to 3.11, *P* = 0.48), tumor location in the middle stomach (OR = 0.85, 95% CI, 0.64 to 1.11, *P* = 0.23) or tumor differentiation (OR = 1.09, 95% CI, 0.80 to 1.48, *P* = 0.58). (Table 2).

**Table 2:** Summary of characteristics between larger IBL group and smaller IBL group.

Characteristics	Studies	Participants (Larger IBL/ Smaller IBL)	Mean Difference/Odds Ratio (95% CI)	Heterogeneity
Baseline information				
Male	4	875/1,618	2.30 [1.17, 4.53]; *P* = 0.02	I^2 ^= 85%; *P* = 0.0002
Female	4	875/1,618	0.43 [0.22, 0.86]; *P* = 0.02	I^2 ^= 85%; *P* = 0.0002
Age, year	2	227/394	0.22 [−1.59, 2.04]; *P* = 0.81	I^2 ^= 0%; *P* = 0.94
BMI, kg/m^2^	3	298/526	0.77 [0.31, 1.22]; *P* = 0.0009	I^2 ^= 0%; *P* = 0.75
Adjuvant chemotherapy	4	875/2,405	1.35 [0.59, 3.11]; *P* = 0.48	I^2 ^= 94%; *P* < 0.00001
Tumor size ≤5 cm	3	849/1,529	0.66 [0.55, 0.78]; *P* < 0.00001	I^2 ^= 0%; *P* = 0.87
Tumor size >5 cm	3	849/1,529	1.52 [1.28, 1.81]; *P* < 0.00001	I^2 ^= 0%; *P* = 0.87
Tumor location-upper	3	674/1,313	2.13 [1.65, 2.76]; *P* < 0.00001	I^2 ^= 0%; *P* = 0.64
Tumor location-middle	3	674/1,313	0.85 [0.64, 1.11]; *P* = 0.23	I^2 ^= 47%; *P* = 0.15
Tumor location-lower	3	674/1,313	0.59 [0.49, 0.72]; *P* < 0.00001	I^2 ^= 0%; *P* = 0.99
Tumor location-whole	3	674/1,313	1.35 [1.07, 1.70]; *P* = 0.01	I^2 ^= 0%; *P* = 0.58
T1–T2	4	875/1,618	0.63 [0.52, 0.76]; *P* < 0.00001	I^2 ^= 3%; *P* = 0.38
T3–T4	4	875/1,618	1.58 [1.30, 1.91]; *P* < 0.00001	I^2 ^= 2%; *P* = 0.38
N0	2	603/1,181	0.70 [0.57, 0.86]; *P* = 0.0007	I^2 ^= 22%; *P* = 0.26
N1–N3	2	603/1,181	1.43 [1.16, 1.75]; *P* = 0.0007	I^2 ^= 22%; *P* = 0.26
Differentiated	2	272/437	1.09 [0.80, 1.48]; *P* = 0.58	I^2 ^= 0%; *P* = 0.48
Undifferentiated	2	272/437	0.92 [0.67, 1.25]; *P* = 0.58	I^2 ^= 0%; *P* = 0.48
Surgery-related information				
Total gastrectomy	3	849/1,529	3.04 [2.47, 3.75]; *P* < 0.00001	I^2 ^= 0%; *P* = 0.73
Partial gastrectomy	3	849/1,529	0.33 [0.27, 0.40]; *P* < 0.00001	I^2 ^= 0%; *P* = 0.73

*Abbreviations: IBL, intraoperative blood loss, mL; T, tumor; N, node; CI, confidence interval.*

### Surgical Information and Short-Term Outcomes

**Figure 2:**
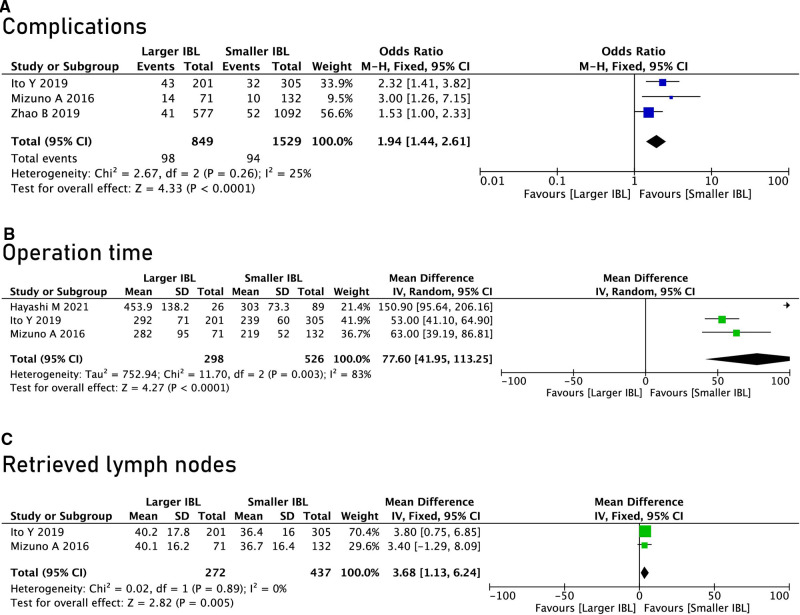
Surgical information and postoperative complications. (**A**) Complications; (**B**), Operation time; (**C**), Retrieved lymph nodes.

The surgical information included the operation time, the extent of resection, the number of lymph node dissections and postoperative complications (including infection, enteroparalysis, venous thrombosis and anastomotic leakage). Through analysis, it could be found that the SIBL group has shorter operation time (OR = 77.60, 95% CI, 41.95 to 113.25, *P* < 0.0001) (Figure 2B), less portion of total gastrectomy (OR = 3.04, 95% CI, 2.47 to 3.05, *P* < 0.00001) (Table 2), and lower postoperative complications (OR = 1.94, 95% CI, 1.44 to 2.61, *P* < 0.0001) (Figure 2A), but the Larger IBL group had higher total retrieved lymph nodes (OR = 3.68, 95% CI, 1.13 to 6.24, *P* = 0.005). (Figure 2C).

### Overall Survival and Disease-Free Survival

**Figure 3:**
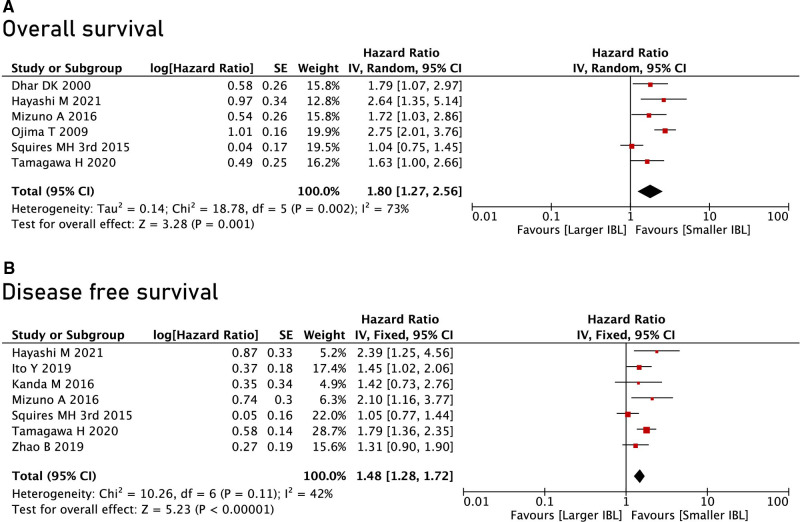
Overall survival and disease free survival between the Larger IBL group and the Smaller IBL group. Note: IBL: intraoperative blood loss, mL.

A total of 9 studies were included in this meta-analysis, of which 6 studies reported OS and 7 studies reported DFS. Through summary analysis, we found that the OS (HR = 1.80, 95% CI, 1.27 to 2.56, *P* = 0.001) and DFS (HR = 1.48, 95% CI, 1.28 to 1.72, *P* < 0.00001) of the SIBL group were better than the LIBL group. (Figure 3).

### Sensitivity Analysis and Funnel Plot

**Figure 4:**
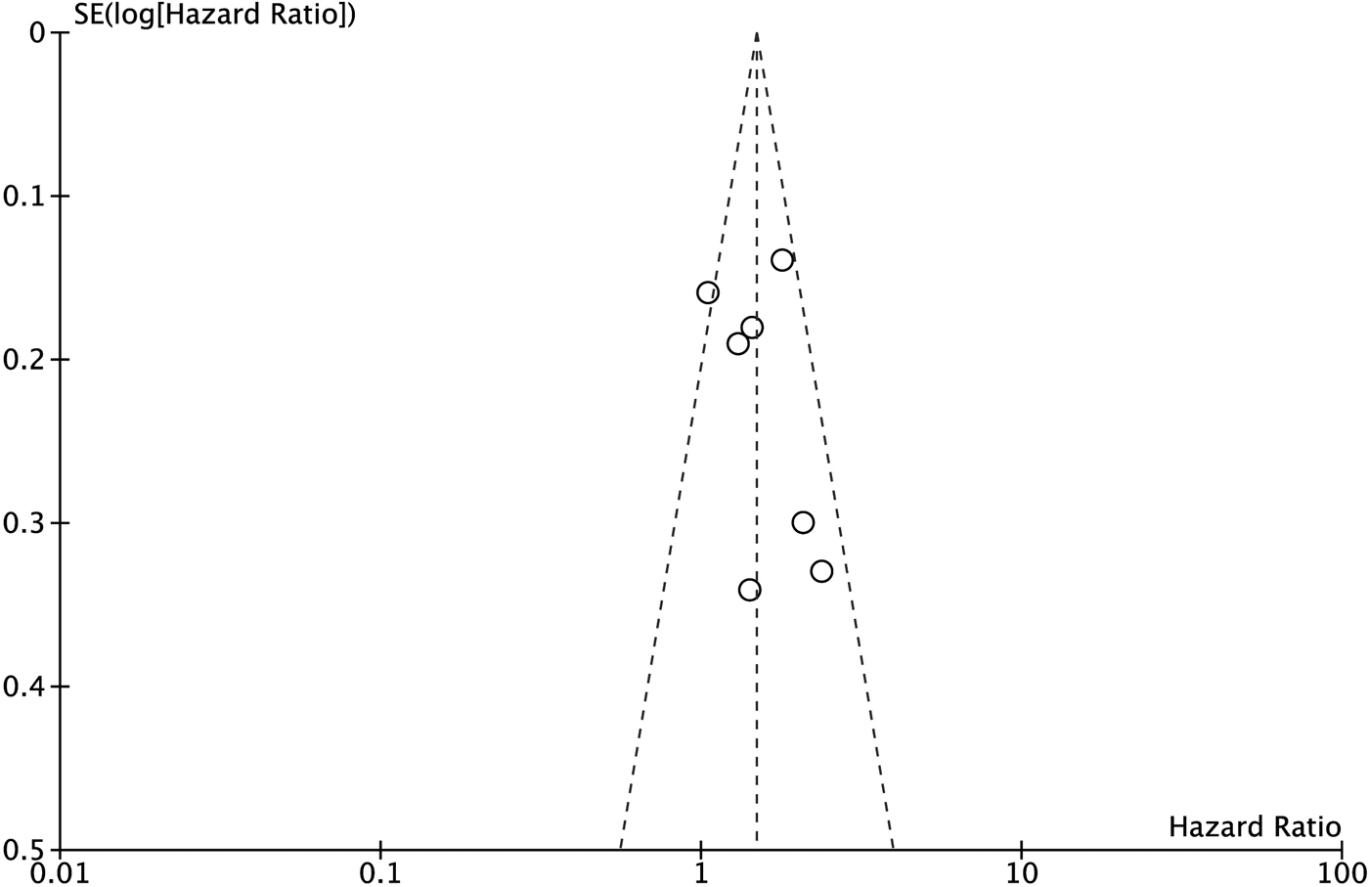
Funnel plots of disease free survival.

Sensitivity analysis was performed by excluding each study one by one, and the results of the excluded study remain unchanged. Moreover, the funnel plot of DFS was analyzed, and the funnel plot was visually symmetrical. (Figure 4).

## Discussion

This meta-analysis included nine studies, including 4653 patients. The SIBL group had shorter operation time, a greater proportion of partial gastrectomy, and a lower incidence of postoperative complications and smaller number of lymph node dissections. Furthermore, the SIBL group had better OS and DFS than the LIBL group.

IBL was often an indicator of the difficulty of gastrointestinal surgery. It has been reported in esophageal cancer and colorectal cancer: Larger IBL were related to increased postoperative complications, and IBL also affected prognosis. In gastric cancer, there were similar reports, but there was some controversy. Zhao et al. (12) reported that IBL did not independently affect the survival of patients after gastrectomy; Hayashi et al. (13) reported that IBL was an independent prognostic factor after gastrectomy; Dhar et al. (19) reported that controlling IBL might improve the survival rate of patients after gastrectomy.

Factors affecting the gastrectomy complications included age, BMI, tumor stage, etc. (27, 28) In this study, large IBL was found to increase complications. The possible reason was that large IBL reduced the body’s immunity, thereby increasing the incidence of complications (29). Therefore, surgeons should operate carefully during surgery to minimize IBL.

In this study, we found that large IBL reduced OS, and the possible reasons were as follows: 1. Large IBL might promote tumor extravasation and hematogenous spread, leading to tumor recurrence and metastasis, especially peritoneal metastasis, thereby affecting the survival. The mechanism might be that the accumulated blood in the peritoneal cavity may provide a favorable microenvironment for the growth of tumor cells. In addition, a large number of angiogenic factors that activate platelets and leukocytes, such as vascular endothelial growth factor (VEGF), could lead to cell proliferation and tumor progression (30). 2. The patient’s immune nutritional status played an important role in tumor immunity (31) and large IBL might hinder anti-tumor immunity, thereby affecting survival (29). 3. Large IBL tended to increase the odds of allogeneic transfusion, which resulted in altered cellular immunity, suppression of natural killer cell activity, and induction of regulatory T cells. These might lead to relative immunosuppression and may allow disseminated or residual microscopic tumor cells to avoid immunodetection, which often led to early recurrence after gastrectomy (32–35).

There was still some important information that could not be included in the meta-analysis due to the limited amount of data. For example, Hayashi et al. (13) reported that the operation time might have an impact on the complications of gastrectomy; Ito et al. (15) reported that the operation method will affect the complications of gastrectomy. Therefore, more relevant information should be reported in future studies.

There were some limitations to this meta-analysis. Firstly, this study included only nine retrospective studies with a total of 4653 patients, with relatively small sample size; secondly, the included studies had different cut-off values for IBL groupings, which might result in heterogeneity; thirdly, most of the included studies were studies on Asian populations and the conclusions may have certain limitations. Finally, some survival data were directly extracted from the Kaplan-Meier survival curve, which may lead to inaccuracies. Therefore, in the future, further high-quality, large-sample, multi-center, and long-term follow-up randomized controlled trials were needed to accurately assess the impact of IBL on short-term outcomes and long-term prognosis after GC surgery.

In conclusion, Larger IBL increased operation time and postoperative complications, and decreased OS and DFS of gastric cancer patients. Therefore, surgeons should be cautious about IBL during operation.

## Data Availability

The original contributions presented in the study are included in the article/Supplementary Material, further inquiries can be directed to the corresponding author/s.
